# Feasibility of Early Intervention Through Home-Based and Parent-Delivered Infant Massage in Infants at High Risk for Cerebral Palsy

**DOI:** 10.3389/fped.2021.673956

**Published:** 2021-07-19

**Authors:** Valentina Menici, Camilla Antonelli, Elena Beani, Alessandra Mattiola, Matteo Giampietri, Giada Martini, Riccardo Rizzi, Alessandra Cecchi, Maria Luce Cioni, Giovanni Cioni, Giuseppina Sgandurra, Caretoy-R Consortium, Claudia Artese

**Affiliations:** ^1^Department of Developmental Neuroscience, IRCCS Fondazione Stella Maris, Pisa, Italy; ^2^Tuscan Ph.D. Programme of Neuroscience, University of Florence, Florence, Italy; ^3^Department of Clinical and Experimental Medicine, University of Pisa, Pisa, Italy; ^4^Neonatal Intensive Care Unit, Pisa University Hospital Santa Chiara, Pisa, Italy; ^5^Division of Neonatology, Careggi University Hospital, University of Florence, Florence, Italy; ^6^Neonatal Intensive Care Unit, Children's Hospital A. Meyer, Florence, Italy

**Keywords:** infant massage, cerebral palsy, early intervention, home-based intervention, family-centered intervention

## Abstract

Infant massage (IM) can be considered an early intervention program that leads to the environmental enrichment framework. The effectiveness of IM to promote neurodevelopment in preterm infants has been proved, but studies on infants with early brain damage are still lacking. The main aim of this study was to assess the feasibility, acceptability and usability of IM, carried out by parents at home, on infants at high risk for Cerebral Palsy. An IM daily diary and an *ad hoc* questionnaire, called Infant Massage Questionnaire Parent-Infant Experiences (IMQPE), were developed. IMQPE consisted of a total of 30 questions, divided into 5 areas. The parents were trained to carry out the IM with a home-based course, conducted by an expert therapist. The intensive IM program was set according to a defined daily length of at least 20 min, with a frequency of at least 5 days per week for a total of 8 weeks. Data collection consisted in the selection of the variables around the characteristics, both of the infants and the mothers, IM dosage and frequency, different body parts of the infants involved and IMQPE scores. Variable selection was carried out by minimizing the Bayesian Information Criteria (BIC) over all possible variable subsets. Nineteen high-risk infants, aged 4.83 ± 1.22 months, received IM at home for 8 weeks. The massage was given by the infants' mothers with a mean daily session dose of 27.79 ± 7.88 min and a total of 21.04 ± 8.49 h. 89.74% and 100% of mothers performed the IM for the minimum daily dosage and the frequency recommended, respectively. All the families filled in the IMQPE, with a Total mean score of 79.59% and of 82.22% in General Information on IM, 76.30% in Infant's intervention-related changes, 76.85% in IM Suitability, 79.07% in Infant's acceptance and 83.52% in Time required for the training. Different best predictors in mothers and in infants have been found. These data provide evidence of the feasibility of performing IM at home on infants at high risk for CP. Study registration: www.clinicaltrial.com (NCT03211533 and NCT03234959).

## Introduction

Infant massage (IM) is defined as any form of systematic tactile stimulation of the infant by human hands, often combined with other types of stimulation such as rocking, kinaesthetic stimulation, talking or eye contact (1). Nowadays this technique is widespread in Neonatal Intensive Care Units (NICU) (2), since it is considered as a valid model of environmental enrichment (3) given its positive effects on the stress of newly-born infants and parent-infant bonding.

A large amount of literature has focused on the effects of IM in infants born preterm without brain lesions. A first meta-analysis and systematic review by Vickers and colleagues (1) analyzed studies that took into account populations composed of infants born preterm and/or low birthweight without any medical complications. The authors highlighted that IM seemed to improve daily weight gain in the treated group compared with controls; a trend in the reduction in terms of length of stay in hospital was also reported even if they argued that there was some methodological bias toward the studies supporting this last finding. However, a meta-analysis by Wang and colleagues (4) confirmed the increased daily weight gain in medically stable massaged preterm populations and supported the hypothesis that massage administration leads to a reduction in the length of stay in hospital. In addition, these authors reported that the possible correlations between IM and neurobehavioral development are still weakly supported in the studies selected due to a lack of consistency, not only in the design of the studies, but also to the lack of follow-up data, to the many differences in the characteristics of the patients and the disparity of treatment protocols.

Updated meta-analyses by Badr et al. (5) and Lu et al. (6) confirmed data on increased daily weight gain in massaged preterm infants medically stable compared with controls. Badr also added data on higher neurodevelopmental scores (assessed with structured developmental scales) in infants that received IM in the NICU compared with controls treated with standard care (7).

In two other systematic review of literature (7, 8) the authors corroborated the hypothesis, with qualitative data, that administering IM to hospitalized preterm infants could have a potential benefit on their growth. In particular, the major findings by Juneau and colleagues in the preterm population treated with massage were a more significant weight gain, less response to pain in terms of less increase in heart rate caused by a painful procedure, more social engagement in parent-infant interaction and a greater score at the Bayley Scale administered at 12 months (7). Álvarez et al. (8) reported that the studies selected in their systematic review supported the benefits of the administration of IM in hospitalized preterm infants in terms of increased vagal activity, increased gastric activity, increased serum insulin; positive effects on the maturation of brain electrical activity and visual function were also reported (9, 10).

Most of the studies carried out on infant massage, as also confirmed by the meta-analysis and systematic reviews available in literature, focused on populations of clinically stable newborns, while a paucity of studies is dedicated to infants with major medical complications. A study by Livingstone and colleagues (11) was developed with the aim to demonstrate the feasibility and safety of IM on infants with complex medical conditions, defined as “fragile infants” and to collect the level of satisfaction of parents reporting positive preliminary results. Significantly, most of the protocols of IM are meant to be applied in the Neonatal Intensive Care Unit (NICU) environment (10, 12–21). The vast majority of them require a nurse or a therapist to massage infants, while in a minority of protocols the mothers were trained to massage their infants (11, 22–28).

A study by Ferber et al. (26) proposed to compare the effects of IM delivered by mothers and by professionals in different populations of preterm infants and found that the expected weight gain was achieved both in the group massaged by the mothers and in the group massaged by a therapist; in addition, a significant decrease in depression symptoms was seen in mothers of preterm infants. This result on the mothers' emotional status was also supported by other studies arguing that anxiety and depression symptoms assessed with self-report questionnaires by the mothers were significantly lower after one or more massage sessions with their infants. This finding was true both for mothers of preterm babies (29, 30) and those of infants born at term (31–33).

All these data contribute to supporting the idea that IM can be proposed as an early intervention (EI) in order to promote physical maturation and neuropsychological development. The increasing number of papers in literature on the beneficial effects of massage on the neurodevelopmental outcome of infants, on the emotional status of mothers in the post-partum period and its positive influence on the quality of parent-infant interaction, as well as the extensive experience in the preterm population, has paved the way for further application of massage. In particular, to our knowledge, no studies have focused on infants at high risk for Cerebral Palsy (CP). Recent literature focused on the sheer importance of early diagnosis and early intervention for this pathological condition that represents the most common physical disability in childhood with a prevalence of 2.1 cases per 1,000 in high-income countries (34). As regards the intervention, it is recommended that it is carried out as early as possible to take advantage of the plasticity of the brain when it is at its maximum level. It should also be intensive, personalized, multi-axial, family-centered and affordable for families and the health service (35). In a recent systematic review of interventions for preventing and treating children with CP, the results of feasibility studies of some EI programs have been included (36). We hypothesized that an innovative application of IM as a home-based intervention administered by the parents, who had previously been adequately trained by a therapist, in the very first months of life after discharge from the NICU in a population of infants at high risk of CP could represent an active standard care of EI. It was included in a larger Randomized Clinical Trial (RCT) comparing the effects of a new technological system, called CareToy-Revised system to the IM (37).

Given the novelty of this hypothesis, feasibility studies of these new proposed approaches such as EI in preterm and at term infants with brain lesions and at risk for CP were required before assessing their effectiveness. CareToy-R Training feasibility had already been assessed by Beani et al. (38). The present study aimed to assess the feasibility, acceptability and usability of IM as a new home EI program.

## Materials and Methods

This feasibility study is part of a larger CareToy-R RCT study described in detail by Sgandurra and colleagues (37). The study was approved by the Pediatric Ethics Committee of Tuscany (84/2017) and registered (NCT03234959) on ClinicalTrials.gov.

In a first stage of the project, families were asked to sign an agreement to participate in an observational phase (http://www.clinicaltrial.gov, NCT03211533) and it was only when the inclusion and exclusion criteria for the infants' enrolment were assessed that the parents were asked to sign and give consent to participate in the interventional trial.

The randomized, evaluator-blinded, multi-center interventional study compared two home-based EIs with two investigative arms (CareToy-R training and Infant Massage) lasting 8 weeks. Eligible infants at high risk of developing CP were randomly assigned to one of these two investigative arms.

This feasibility study focuses on IM provided for an intensive and continuous period of time to infants at high risk of CP by their parents.

### Participants

The participants of the CareToy-R study were recruited by a child neurologist in the NICUs or on the occasion of neurodevelopmental follow-up visits in 3 University Hospitals in Tuscany (Italy): the Meyer Children's Hospital and the Careggi General Hospital, in Florence, and the Santa Chiara Hospital in Pisa. The intervention study was managed by clinical and rehabilitation staff of Developmental Neuroscience, IRCCS Fondazione Stella Maris, Pisa.

The subjects deemed eligible for the CareToy-R study were both preterm or full-term infants with brain lesions as reported by Neonatal Brain Ultrasonography (US) or Magnetic Resonance Imaging (MRI). Infants with polymalformative syndromes, severe sensory impairments (retinopathy of prematurity grade > II, deafness or blindness) and cerebral malformations were excluded. The selection process included a clinical and neurological examination of infants at risk at 3 months corrected age, using the General Movements assessment (GMA) and the Hammersmith Infant Neurological Examination (HINE).

The subjects were selected when atypical patterns at the GMA and/or specific neurological signs at the HINE were observed.

When the infants selected achieved pre-established motor skills (starting from the initial head control) defined on the basis of the cut-off scores of the Ages & Stages Questionnaire, they were randomly allocated to one of the two investigative arms (CareToy-R Training or the IM intervention) of the RCT.

Recruitment for this preliminary study on feasibility started once the approval of the Ethics Committee was obtained. This feasibility study involved those infants randomly assigned to the IM intervention.

### Study Design and Procedures

The minimum sample size for the IM group was set at 19 infants. Recruitment started in September 2017 and ended in June 2020.

During the intervention, infants continued to benefit from the standard care (SC) provided by the National Health System (NHS) and parents were asked to complete a diary to define and quantify the content of the SC.

A child neurologist and a therapist evaluated infants at the following times:

- T0 (baseline), the week before starting IM or CareToy-R Training interventions
- T1 (primary endpoint), a week after the end of the intervention
- T2, 8 weeks after the end of the intervention
- T3 (last follow-up), at 18 months corrected age of the infant

Standardized clinical tools and questionnaires were administered at all time point assessments. The primary outcome measure of the RCT study was the Infant Motor Profile (IMP) (39, 40), a video-based assessment of motor behavior in infancy that can also be used to assess infants at high risk of CP (41, 42). Secondary measures included Peabody Developmental Motor Scales—Second Edition (PDMS-2) (43, 44), Bayley Scales of Infant Development Cognitive subscale (BSID-III) (45), standardized video-recordings of parent-infant interaction (46, 47), Teller Acuity Cards® (48) and Actigraphic analysis (Motionlogger Microwatch) (49). Moreover, parents were also asked to fill in the BSID-III Social-Emotional Scale (50) and the Parenting Stress Index questionnaire (PSI) (51).

After the intervention period, families were asked to fill in a questionnaire on the feasibility of their intervention: “CareToy-Revised Questionnaire Parent-Infant Experiences” (38) and “Infant Massage Questionnaire Parent-Infant Experiences” (see details below), respectively.

It should be noted that some post-intervention evaluations have been delayed due to the COVID-19 pandemic breakdown.

### Intervention

#### Infant Massage Intervention

We proposed massage as a home-based early intervention to be provided by parents who had been previously trained by a therapist. The intervention lasted 8 weeks and parents were asked to massage their infants at least 20 min a day (in one or more daily sessions) for a minimum of 5 days per week. They were also asked to write information in a daily diary about the duration of each IM session and the sequences of movements provided each time.

The IM course was provided at home and organized in 5 sessions of 1 h each, scheduled every 7–10 days.

During the IM session the therapist first assisted the parents in creating the optimum setting so as to derive the greatest benefit from the interaction with their infant. The therapist then performed massage sequences on a doll while the parents imitated the sequences of massage on their infants.

The order of the IM sequences taught was not mandatory; the therapist explained and showed all the sequences in different orders depending on the tolerability of the infant and his/her response to the massage. Once all the sequences had been illustrated (sequences: legs and feet, stomach, chest, arms and hands, face and back), the parents were invited to personalize the order of the massage sequences according to the infants' preferences.

A team of clinical and rehabilitative professionals (mainly child neurologists and therapists) was available throughout the duration of the study to answer any requests from the families regarding the IM intervention. The therapist, in some cases, was available for assistance and, on occasion, if necessary, sent some explanatory videos or scheduled video calls with families to resolve doubts about the massage sequences.

### Outcome Measures

The feasibility of IM was evaluated according to three different thematic areas which focused on the intervention, on the study design and its procedures and on the acceptability and usability of the intervention from the parents' point of view.

For each area a general main question was formulated, and a multi-dimensional answer was elaborated on the basis of defined criteria that had to be fulfilled.

The feasibility criteria for this study were taken from recommendations that can be found in literature (52–55).

#### Feasibility of the Intervention

The main question asked regarding this point, was “Is the intervention suitable and acceptable for the participants?” The answer was formulated on the basis of the parents' daily diaries on the intervention and these measures were taken into account:

- Intervention compliance and motivation: difference between IM (days and hours) requested and total IM administered (days and hours).
- Intervention adherence: total number of days in which at least 20 min of IM was performed.
- Intervention and participation in appointments: number of lost SC appointments during the IM intervention due to tiredness or physical discomfort of the infant.

Definitions and measurements for the feasibility criteria of this intervention can be found in Table 1.

**Table 1:** Feasibility, usability, and acceptability criteria.

	**Main question**	**Areas**	**Definition**	**Feasibility question**	**Feasibility criterion for success**	**Measurement**
Feasibility of intervention	“*Is the intervention suitable and acceptable for the participants?”*	Intervention compliance and motivation	Parents motivation and compliance to perform infant massage	Are participants compliant and motivated to perform training intervention?	Difference between IM (days and hours) requested and total Infant Massage administered (days and hours)	Parents' daily massage diaries
		Intervention adherence	The extent to which the families followed the instructions for administering the massage provided for in the study	Do participants perform at least 20 min of infant massage per day?	Total number of days in which a IM of at least 20 min was performed	Parents' daily massage diaries
		Intervention and participation in appointments	To evaluate whether infant massage fitted in with the families' daily play and rehabilitation activities (Standard Care)	Does the infant massage fit in with the families' daily play and rehabilitation activities?	Number of missed SC appointments during the IM intervention due to tiredness or physical discomfort of the infant	Parents' daily appointments diaries
Feasibility of study and its procedure	“*Is the intervention suitable and acceptable for the participants?”*	Participation willingness	Percentage of families that accepted to participate in the study	What is the participation rate?	At least 80% of eligible participants agreed to join the project	Caretoy database
		Participation rates	Percentage of dropouts	Do all eligible participants agree to perform the infant massage intervention?	80% of participants who gave consensus participated in the study	Caretoy database
		Data loss in the follow-up	Percentage of data recorded on time at all timepoints	Can all data be collected without any problems?	90% of the outcome measures were collected	Caretoy database
		Assessment time scale	Time required for collecting all the outcome measures at each timepoint	Can follow-up data be collected within a week after the training period?	Time from end of training period to first follow-up data collection	Recorded data of the beginning and the end of the infant massage (daily massage diaries) and data of assessments
		Assessment procedures	Number of patients who failed to complete the outcome measures during follow-up.	Is the loss to follow-up acceptable?	Less than 20% of participants failed to complete outcome measures on all follow-up assessments	Collection of data report by examiners
Acceptability and usability of the intervention from parents' point of view	“*To what extent is the intervention acceptable and usable according to the participants?”*	*Ad hoc* questionnaires	An *ad hoc* questionnaire on the standard definition of acceptability and usability was compiled	Is the intervention acceptable and usable for participants?	At least 65% of total score in IMQPE questionnaire was achieved	Results of IMQPE questionnaire

#### Feasibility of the Study Design and Its Procedures

The main question asked regarding this point, was “Is the intervention suitable and acceptable for the participants?” To answer this question, data included in the RCT study database were used and the following measurements were analyzed:

- Participation willingness: percentage of families that agreed to participate in the study.
- Participation rate: percentage of dropouts (percentage of infants who abandoned the 8 week intervention).
- Data loss in the follow-up: percentage of data recorded on time at all timepoints.
- Assessment time scale: time required for collecting all the outcome measures at each timepoint.
- Assessment procedures: number of patients who failed to complete the outcome measures during follow-up.

Definitions and measurements for the feasibility criteria for this intervention can be found in Table 1.

#### Acceptability and Usability of the Intervention From the Point of View of Parents

As far as this point is concerned, the main question was “To what extent is the intervention acceptable and usable according to the participants?” To answer this question, an *ad hoc* questionnaire on the standard definition of acceptability (56, 57) and usability (58–60) criteria was compiled. The pivotal role of parents in providing IM was taken into account to create the ‘Infant Massage Questionnaire Parent-Infant Experiences (IMQPE)' as well as for the questionnaire created for the CareToy-R Training (38).

All families were asked to reply to the IMQPE in order to understand and collect their opinions on IM in the post-intervention period.

There are 30 questions in the IMQPE, which are divided into 5 areas (with 6 questions for each area with a maximum total score of 150 points): (1) General information on IM, (2) Infant's intervention-related changes, (3) IM suitability, (4) Infant's acceptance, and (5) Time required for the training. Questions were measured with a 5-point Likert scale [families were instructed to choose the most appropriate response, ranging from “totally agree” (score 5) to “strongly disagree” (score 1)] and with some open questions in which parents could express their thoughts or add qualitative comments.

### Data Collection and Statistical Analysis

Dedicated Case Report Forms (CRF) were developed in order to collect both the infant's and mother's demographic characteristics as well as the Parents Stress Index questionnaire scoring results.

For IM data, parents were asked to fill in a daily diary with a detailed description of the IM sessions in terms of body regions massaged and duration of the sequences provided.

At the end of each intervention, the information reported in the parents' diaries was digitalized in a spreadsheet by filling in the following items: number of times parents performed a sequence dedicated to a particular body region (legs and feet, arms and hands, stomach, chest, face, and back) during the 8 weeks of intervention, number of times these sequences were performed considering only the period after training (in this case the variable was defined “Post Training” or PT), duration of the IM expressed in minutes per day and total amount of hours spent delivering IM during the 8 weeks period. For each item, the mean and the standard deviation were calculated.

Subsequently, the IMQPE questionnaire was administered by a psychologist via phone call to the parent responsible for the massage. This procedure made it easier for families to understand all the questions since they could ask the interviewer directly for clarification and they could feel free to express their own opinions.

The IMQPE questionnaire scores were also reported in a spreadsheet, as well as the relative percentages, calculated with respect to the total of the questions (Total score) and to each area.

For each item, a linear regression model was fitted after a variable selection step. The best model was found by minimizing the BIC (Bayesian Information Criteria) over all possible predictors subsets. The analysis was carried out with R-version 4.0.1. Significance level was set to 0.05.

## Results

### Participants

Nineteen infants were allocated to the IM intervention group and all the families completed all the assessments planned for the study and filled in the IMQPE questionnaire.

The study population was composed of 10 males, 9 females; 6 single-born subjects, 13 with siblings and 6 of the latter had a twin.

Thirteen infants were preterm (2 late preterm, 6 very preterm, and 5 extremely preterm) and 6 were born at term.

All the subjects had a brain injury on early neuroimaging: 4 of them were affected by an hypoxic-ischemic encephalopathy (HIE); 6 of them suffered an intraventricular hemorrhage from grade II to grade IV (IVH) (1 subject with grade II, 3 subjects with grade III, 2 subjects with grade IV); 7 of them reported a periventricular leukomalacia (PVL) and 2 subjects had an history of perinatal stroke.

The mean age of the infants at T0 assessment was 4.83 ± 1.22 months (range 3.00–6.74 months). In all the families, IM was administered by the mothers, whose mean age was 33.16 ± 7.03 years (range 19–45 years). 68% of the mothers were Italian and 32% were of foreign origin (2 Moroccans, 1 Albanian, 1 Macedonian, 1 Russian, and 1 Chinese). Families participating in the study lived in different Regions of Italy. The mean distance from IRCCS Fondazione Stella Maris was 167.64 ± 225.82 km, ranging from 12 km (Livorno, the nearest place) to 993 km (Santa Maria di Leuca in Puglia, the farthest).

The demographic characteristics of the mothers and infants can be found in Table 2.

**Table 2:** Sample characteristics.

**Sample characteristics**	
**Infants' characteristics**	
Infants' sex: *n* (%)	Male: 10 (53%)
	Female: 9 (47%)
Mean gestational age ± SD (range) (weeks)	31.84 ± 5.90 (24^+0^-40^+10^)
Mean infant age ± SD (range) at T0 (months)	4.83 ± 1.22 (3.00–6.74)
Brotherhood: *n* (%)	13 siblings (68%)
	6 only-child (32%)
Twin: *n* (%)	6 twins (32%)
Type of lesion: *n* (%)	Hypoxic-ischemic encephalopathy: 4 (21%)
	Intraventricular Hemorrhage: 6 (32%)
	Periventricular Leukomalacia: 7 (37%)
	Stroke: 2 (10%)
**Mothers' characteristics**	
Mean mothers' age ± SD (range)	33.16 ± 7.03 (19.00–45.00)
Mothers ‘emotional status nationality: *n* (%)	Italian: 13 (68%)Foreign: 6 (32%)
Mothers' employment: *n* (%)	Employed: 12 (63%)
	Unemployed: 7 (37%)
PSI subscales score: mean ± DS	PSI PD: 30.00 ± 10.33PSI-CDI: 21.31 ± 7.81PSI-DC: 25.10 ±9.35PSI-TS: 76.42 ± 22.70
Mothers' educational level according to the ISCED: *n* (%)	Level 1–2: 5 (26.31%)Level 3: 8 (42.10%)Level 6–7: 3 (15.79%)Level 8: 3 (15.79%)

*PSI, Parent Stress Index; PD, Parental Distress; P-CDI, Parent-Child Dysfunctional Interaction; DC, Difficult Child; TS, TOTAL SCORE; ISCED, International Standard Classification of Education*.

### Feasibility of the Intervention

The feasibility criteria were met as follows:

✓ Intervention compliance and motivation: IM was performed in all cases above the minimum requested by the study. 89.47% of mothers performed IM for more than the minimum number of hours recommended (i.e., 13.33 h) for the study with a total range of IM between 13.55 and 40.08 h. Only in two cases was the total amount of IM lower, 8.63 and 11.42 h. Infants received a mean total IM of 21.04 ± 8.49 h.
✓ Intervention adherence: All mothers massaged their infants at least 5 days per week, but four of them in some days were not able to massage the infant for at least 20 min every day.
The daily mean length of massage administration was 27.79 ± 7.88 min.
✓ Intervention and participation in appointments: mothers were able to organize the IM in their daily routine and integrate it with SC (visits, physiotherapy, follow-up).

In particular, 84% of infants attended motor therapy sessions with the following frequency: 4 infants were monitored with one session every 2 weeks, 3 infants attended the rehabilitation treatment once a week, 6 infants twice a week, and 4 infants three times a week.

Moreover, all the infants had monthly follow-up visits, pediatric visits, and some of them received neurodevelopmental assessments in third level centers. Most of them are also subject to mandatory vaccinations according to the NHS.

### Feasibility of the Study and Its Procedures

The feasibility criteria of this study were fulfilled as follows:

✓ Participation willingness: all the families accepted the invitation to participate in the study when asked.
✓ Participation rates: all participants completed the intervention.
✓ Data loss in the follow-up: it was possible to record all the data of all outcome measures and there were no missing data
✓ Assessment time scale: follow-up measurements of 74% of participants were collected within 1 week after the end of the intervention period (range 0–7 days after the end of IM). 26% follow-up measurements were collected between 8 and 17 days after the training because of the COVID-19 pandemic breakdown, the distance from the center and the holiday period (mainly Christmas and summer holidays). The follow-up at T1 was carried out after a mean of 6.72 ± 5.13 days from the end of the IM period.
✓ Assessment procedures: all participants completed the assessment at all the timepoints.

### The IMQPE Questionnaire

All 19 families accepted to fill in the questionnaire and the semi-structured interview was carried out by a psychologist of the NICU of Santa Chiara University Hospital in Pisa.

All participants reported a total score above 102 points (68.00%) at the IMQPE, with a range of 102–137 points and a mean total score of 119.39 ± 9.27 points (79.59%).

Regarding the five sections scores, in “General information on IM” the range of the raw scores was between 20 and 30 points (mean of 83.52%); in “Infant's intervention-related changes” the range of the raw scores was between 16 and 30 points (mean of 76.30%); in “IM suitability” the raw score was between 19 and 26 (mean of 76.85%); in “Infant's acceptance” the raw score was between 15 and 30 points (with a mean of 79.07%) and in “Time required for the training” it was between 20 and 29 points (with a mean of 82.22%).

**Figure 1:**
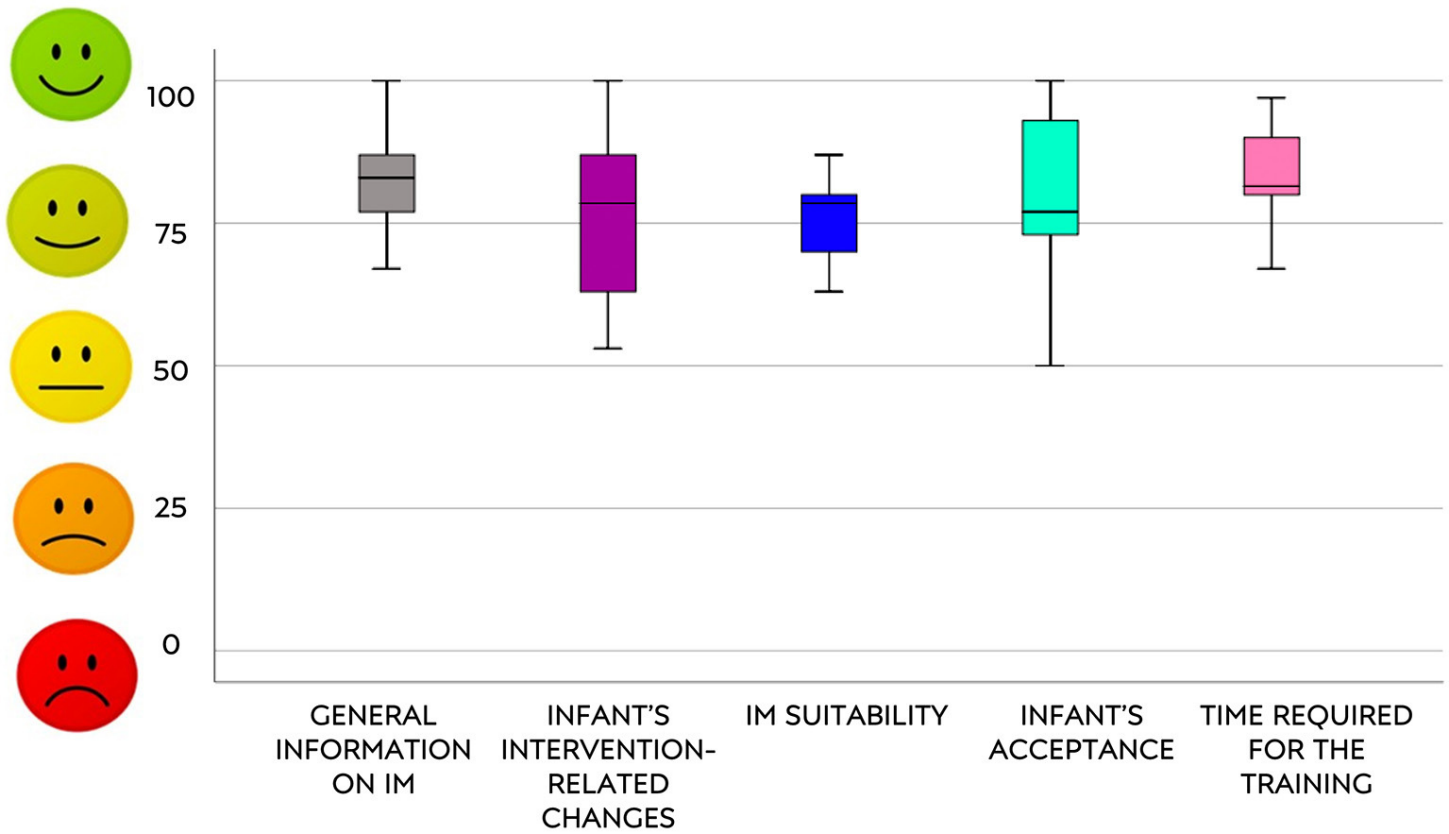
Answers of IMQPE sections.

**Figure 2:**
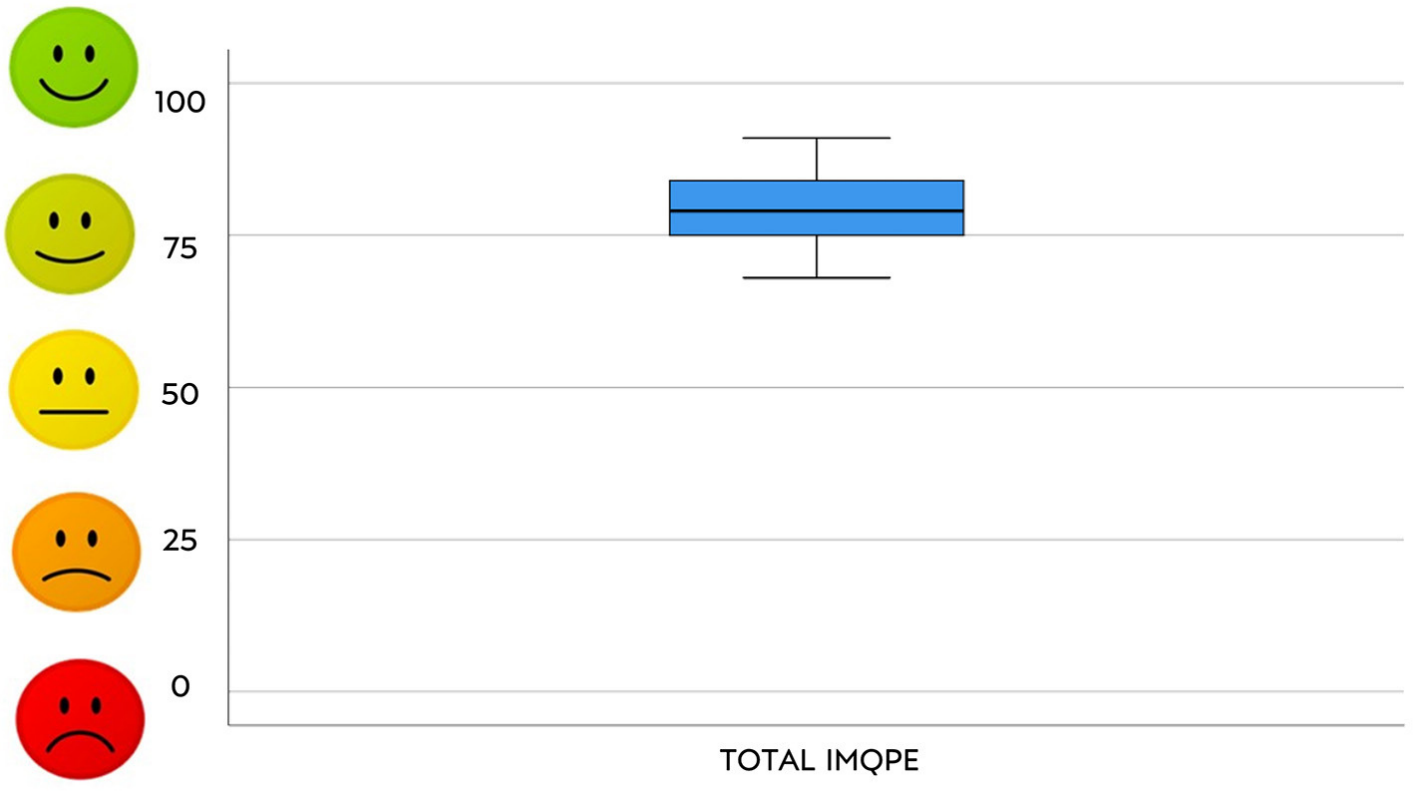
Total answers to the IMQPE.

Median and 95% confidence interval of percentages scores in the questionnaire (both total and section scores) are shown in Figures 1, 2.

### Relationship Between Infant and Mother Characteristics

Considering the mothers' characteristics and the amount of IM administration (mean daily and total hours of IM and frequency of execution of each sequence during the entire intervention and post training) the best predictive significant models were found between nationality of the mother and the frequency of the provided Arms and Hands sequences, post training Arms and Hands sequences and post training Legs and Feet sequences. The results are shown in Table 3.

**Table 3:** Mothers' characteristics and the amount of IM.

	***R*^**2**^**	**Estimate**	**SE**	***t*-value**	***p***
**PT Legs and Feet**					
Mothers' nationality (Italian)	47.50%	−0.67	0.29	−2.29	0.04
**Arms and Hands**					
Mothers' nationality (Italian)	28.80%	−9.53	3.63	−2.62	0.02
**PT Arms and Hands**					
Mothers' nationality (Italian)	33.10%	−9.80	3.38	−2.90	0.01

*SE, Standard Error; PT, post training*.

The mother's nationality factor predicted three variables of model, as reported in Table 4.

**Table 4:** Variables of model between mothers' characteristics and the amount of IM.

	**N^**°**^ of model**	**Variables**
**Mothers' nationality**	3	PT Legs and Feet
		Arms and Hands
		PT Arms and Hands

*PT, post training*.

Considering the characteristics of the infants and the amount of IM administration (mean daily and total hours of IM and frequency of execution of each sequence during the entire intervention and post training) the best predictive models are shown in Table 5.

**Table 5:** Infants' characteristics and the amount of IM.

	***R*^**2**^**	**Estimate**	**SE**	***t*-value**	***p***
**Mean daily IM**					
Twins	60.40%	−11.66	3.79	−3.08	<0.01
Gestational age		−1.41	0.66	−2.15	0.05
Lesion stroke		11.53	5.22	2.21	0.05
**Total Hours IM**					
Siblings	45.30%	8.99	3.88	2.32	0.03
Gestational age		−0.71	0.34	−2.08	0.05
Twins		−14.83	4.37	−3.39	<0.01
**Arms and Hands**					
Twins	30.50%	−10.48	4.17	−2.51	0.02

*SE, Standard Error; IM, Infant Massage*.

The characteristics of being a twin predicted three variables of model, gestational age predicted two variables, being siblings and having a stroke lesion predicted one variable each, as reported in Table 6.

**Table 6:** Variables of model between infants' characteristics and the amount of IM.

	**N^**°**^ of model**	**Variables**
Twins	3	Mean daily IM
		Total Hours IM
		Arms and Hans
Gestational age	2	Mean daily IM
		Total Hours IM
Lesion stroke	1	Mean daily IM
Siblings	1	Total Hours IM

*IM, infant massage*.

### Relationship Between IMQPE Questionnaire and IM

The best significant predictor models considering the results of the IMQPE questionnaire and the amount of IM administration (mean daily and total hours of IM and frequency of execution of each sequence during the entire intervention and post training) are shown in Table 7.

**Table 7:** IMQPE questionnaire and the amount of IM.

	***R*^**2**^**	**Estimate**	**SE**	***t-*value**	***p***
**Mean daily IM**					
Infant's acceptance	20.70%%	0.84	0.41	2.05	0.06
**Arms and Hands**					
Infant's acceptance	49.40%	1.91	0.54	3.54	<0.01
Infant's intervention related changes		−1.35	0.53	−2.55	0.02
**PT Arms and Hands**					
Infant's acceptance	29.10%	1.25	0.052	2.40	<0.03

*SE, Standard Error; IM, infant massage; PT, post training*.

The “Infant's acceptance” score predicted three variables of the model, the “Infant's intervention-related changes” score predicted one variable and “General information on IM” score predicted one variable, as reported in Table 8.

**Table 8:** Variables of model between IMQPE questionnaire and the amount of IM.

	**N^**°**^ variables of model**	**Variables**
Infant's acceptance	3	Mean daily IM
		Arms and Hands
		PT Arms and Hans
Infant's intervention related changes	1	Arms and Hands
General information on IM	1	Arms and Hands

*IM, infant massage; PT, post training*.

### Relationship Between IMQPE Questionnaire and Mothers' and Infants' Characteristics

Considering the PSI scores of the mothers and the IMQPE questionnaire scores, the best predictor factor was found between the Parental Distress subscale and the “Infant intervention-related changes” score, as shown in Table 9.

**Table 9:** IMQPE questionnaire and mothers' characteristics.

	***R*^**2**^**	**Estimate**	**SE**	***t*-value**	***p***
**Infant's intervention related changes**					
PSI-PD	44.60%	−0.72	0.23	−3.05	<0.01

*SE, Standard Error; PSI, Parent Stress Index; PD, Parental Distress*.

The “Infant intervention-related changes” area predicted one variable of model, as reported in Table 10.

**Table 10:** Variables of model between IMQPE questionnaire and mothers' characteristics.

	**N^**°**^ variables of model**	**Variables**
Infant's intervention related changes	1	PSI-PD

*PSI, Parent Stress Index; PD, Parental Distress*.

The results of the IMQPE questionnaire and the infants' characteristics are reported in Table 11.

**Table 11:** IMQPE questionnaire and infants' characteristics.

	***R*^**2**^**	**Estimate**	**SE**	***t*-value**	***p***
**IM suitability**					
Twins	48.90%	2.46	1.01	2.44	0.03
**Time required for the training**					
Siblings	56.20%	−3.09	1.00	−3.08	<0.01
Lesion IVH or PVL		−6.58	2.54	−2.59	0.02

*SE, Standard Error; IM, infant massage; IVH, intraventricular hemorrhage; PVL, periventricular leukomalacia*.

The characteristic of being a twin predicted one variable of the model, as well as brotherhood and IVH or PVL lesion. The results were reported in Table 12.

**Table 12:** Variables of model between IMQPE questionnaire and infants' characteristics.

	**N^**°**^ variables of model**	**Variables**
Twins	1	IM suitability
Siblings	1	Time required for the training
Lesion IVH or PVL	1	Time required for the training

*IM, infant massage; IVH, intraventricular hemorrhage; PVL, periventricular leukomalacia*.

## Discussion

To our knowledge, this is the first study in literature which assesses the feasibility, the acceptability and the usability of IM as an EI program dedicated to a population of infants at high risk for CP, delivered at home by their parents, who had been previously trained by an expert therapist.

The home-based nature of the IM early intervention, ongoing for 8 weeks, significantly differs from the other programs proposed in the vast majority of the studies in literature. These studies mainly investigate the short-term clinical benefits of a usually brief cycle of massage administration in the NICU before discharge from hospital (1, 4, 5, 7, 8).

This study population is also different from the previous studies as it involves both preterm and at term born infants with a brain injury and atypical patterns at standardized neurological examinations with a consequent high risk for developing a CP.

Our protocol proposal combines most of the key factors required for an EI program according to the most recent literature on CP (35, 61–63).

We expect a positive impact of this intervention on neurodevelopmental outcome of infants at high risk for CP. Previous studies have suggested the potential value of IM as an intervention in the framework of environmental enrichment (64, 65). Specifically, a sensitive parent-infant bonding and a stimulating home environment have been associated with an effective shaping of cortical plasticity and with a better neurodevelopmental outcome in preterm infants. In this framework, IM seems to be characterized by some of the key features of a successful early developmental intervention program for preterm babies, being based on parents' empowerment and can be performed in the NICU as well as at home, after the hospital discharge. However, its efficacy has to be proven also for a population at high risk for CP that may include pathological conditions other from prematurity alone (66).

We proposed an intensive family-centered approach, where the IM would be delivered by the parents at home, even by those living far from our clinical center, for 8 weeks. The choice that the IM intervention could be delivered directly by the parents is consistent with literature that underlines the importance of taking into account the parents' emotional status and the beneficial effects of massage on depression and anxiety symptoms, mainly in mothers after giving birth (29, 30, 32, 67). Moreover, the home-based nature of this intervention has allowed the families to personalize the administration of the massage, albeit in the context of the instructions provided by the therapist regarding the duration and the frequency of the sessions. Furthermore, the parents had the possibility to choose the best timing and the most suitable sequences of IM according to the infants' and parents' preferences.

The feasibility analysis of this type of EI is very innovative. Most of the feasibility studies available in literature are mainly focused on the feasibility of home-rehabilitation with technologies (38, 68, 69) so in this study the feasibility evaluation criteria were customized to a home intervention conducted without the use of technological tools (52–55).

As regards the feasibility of the study and its procedures, the data collected supported the high rate of acceptance of the general RCT project since all the families who were asked to join agreed to participate.

All the families completed the protocol of intervention participating in all the scheduled follow-up visits. No dropouts were reported. Even if the IM training and delivering was proposed both to mothers and fathers, only mothers conducted the intervention. This may be due to the higher availability of the mothers, who are usually at home on maternity leave. The mothers' intervention compliance and the motivation were high. All the mothers, indeed, administered a daily mean of IM (in minutes) above the minimum duration requested; most of them administered the IM more than 5 days per week which was the minimum weekly frequency required. We have also found some interesting best predictors of the mothers' characteristics. The foreign mother provided Legs and Feet and Arms and Hand sequences more frequently.

Moreover, the parents of twins provided less IM in terms of mean daily sessions and total hours of IM conducted; in particular the Arm and Hands sequence was the least performed by this group. However, they were related to higher values of IM suitability, and not to encountering difficulties in running sequences in both infants. By contrast, the mothers with only one child performed more IM in terms of total hours than parents with more than one child. We can hypothesize that parents with only one child may find it easier, in terms of time, to include the massage among the daily activities of the family. Furthermore, the lower the value of gestational age, the higher the amount of IM carried out. It could be related to the mothers' greater interest in having tactile contact with their infants, as they usually stay until the term period in the NICU.

Furthermore, thanks to the description reported in the parents' daily appointments diaries, we found that the mothers were able to combine the IM administration with SC (visits, physiotherapy, follow-up). The mothers did not observe their infants suffering from fatigue after the massage and they were therefore able to participate in the rehabilitative sessions provided by the NHS even on the same day as the IM.

As regards the type of lesion the infants presented, stroke had been identified as a predictive factor for receiving a greater mean daily amount of IM. The interpretation of these data is not simple on the basis of the available literature, given the lack of studies on the population at neurological risk. We can speculate that this population had fewer medical complications related to their neurological illness, so they were possibly willing to receive IM sequences. For the IVH or PVL lesion types, the mothers needed to spend a greater amount of time, even if they did not carry out a higher amount of IM sequences.

More sessions for a potential higher neurological impairment of these infants may be necessary, even if this hypothesis needs to be confirmed in the clinical RCT study.

As regards the acceptability ad usability investigated by means of the IMQPE, very interesting results have been obtained. In particular, in the “General information on IM” area the parents reported a higher score since they widely appreciated this kind of intervention from many points of view, considering it useful in enhancing and promoting interaction and attunement with their infants.

The lowest score was reported in the “Infant intervention-related changes” area, although in the Likert scale, the score obtained highlighted how a certain degree of change was perceived by the parents due to the IM intervention. However, considering the PSI subscale scores, a higher score in the mothers Parental Distress subscale was predictive of a lower perception of changes in the infant which could be related to the emotional difficulties in perceiving such changes. High levels were found for the “IM suitability,” as the mothers had not encountered any difficulties in carrying out the sequences and generally did not need any additional assistance from the therapists. High scores were achieved also in the areas of “Infant's acceptance” and “Time required for the training.” Questions related to the infants' acceptability of IM and the role of the parent while performing the intervention were included in these areas. This could highlight that the infants had appreciated the IM and the mothers (who had conducted the IM) felt free and confident with this approach; moreover, most of them reported that the time required to dedicate to IM was adequate. It should also be pointed out that the higher levels of acceptance on the part of the infants were related to the massage of Arms and Hands (during the entire intervention and in the post training period) even if lower levels of changes in the infants were perceived. However, the results of the standardized clinical outcome measures in the RCT can provide evidence of the effectiveness of the IM. Finally, from the general analysis of the interviews conducted with the mothers, it emerged that a very good experience was had by all. The mothers recounted how they had felt a deep sense of involvement in IM practice and a sense of satisfaction in sharing IM sequences with their infants and found the approach calming, pleasant, beautiful, engaging, and relaxing. Furthermore, they reported that the IM experience was an occasion to get to know each other better.

In the present study, there are some limitations that need to be discussed. First of all, the answers given in the questionnaire could be overestimated because the mothers when interviewed were not blind to the intervention but were active actors and fully devoted to delivering it. In addition, the interviews done through phone calls could not allow an objective evaluation of the mothers. Furthermore, some results are difficult to interpret, due to the lack of clinical measurements that may allow an objective evaluation of their changes and of IM effectiveness, for example those comparing the perception of changes with the real clinical changes, or the impact of IM on Parental Stress. Moreover, the heterogeneity of the brain injuries without a stratification and the small sample size represents an important limitation and requires caution in results interpretation and in generalizing the feasibility of this approach to the large CP population; for this reason, we suggest considering our study as a pilot study. Another limitation that we can point out is the lack of information on the parental coping with respect to the NICU communication on the neurological risk of their infants. The literature supports the importance of an early diagnosis and of effective communication strategies for diagnosis disclosure to the parents (70, 71). This aspect could also be considered prog-nostic for family acceptance of an EI proposal, but, unfortunately, we did not collect systematically information concerning this specific issue, and therefore correlations with the feasibility indices could not be analyzed.

Finally, a cost-effectiveness detailed analysis of the study was not carried out. This analysis is crucial to assess the real possibility of using IM to reduce the costs of health services and to offer a relatively inexpensive home-intervention.

Besides these limitations, the current study, and the previous publication of Feasibility of the other arm of the study on CareToy-R training (38) lay the groundwork for the feasibility of two active EI home based programs in infants at high risk for CP. Moreover, the innovative use of standard criteria to assess the EI feasibility could be useful to encourage, and the compare future studies. The parent's participation and commitment and the feasibility of EI programs at home are absolutely crucial for home-based interventions.

## Data Availability Statement

The raw data supporting the conclusions of this article will be made available by the authors, without undue reservation.

## Ethics Statement

The studies involving human participants were reviewed and approved by Paediatric Ethics Committee of Tuscany. Written informed consent to participate in this study was provided by the participants' legal guardian/next of kin.

## Author Contributions

GS and GC conceived the idea for this original research and all other authors contributed to the conception and the design of the study. RR, AC, MC, and MG carried out the enrolment of all infants for the study. RR did the baseline neurological assessment and eligibility evaluation. VM, EB, and GS designed and realized the questionnaire. VM and GM performed the IM training to the parents. AM administered the IMQPE questionnaires. CA, VM, GS, and GC conceived and prepared the manuscript. All the authors read, critically revised, and approved the final manuscript.

## Conflict of Interest

The authors declare that the research was conducted in the absence of any commercial or financial relationships that could be construed as a potential conflict of interest.

## The Caretoy-R Consortium Members

Claudia Artese^1^, Veronica Barzacchi^2^, Marta Cervo^3^, Carlo Dani^1^, Paolo Dario^4^, Marco Di Galante^5^, Ugo Faraguna^5, 6^, Patrizio Fiorini^7^, Viola Fortini^3^, Simona Giustini^1^, Clara Lunardi^1^, Irene Mannari^4^, Martina Maselli^4^, Martina Orlando^2^, Letizia Padrini^7^ and Filomena Paternoster^7^

^1^Division of Neonatology, Careggi University Hospital of Florence, Florence, Italy

^2^Department of Developmental Neuroscience, Istituto di Ricovero e Cura a Carattere Scientifico (IRCCS) Fondazione Stella Maris, Pisa, Italy

^3^Rehabilitation Department, Children's Hospital A. Meyer, Florence, Italy

^4^The BioRobotics Institute, Scuola Superiore Sant'Anna, Pisa, Italy

^5^Istituto di Ricovero e Cura a Carattere Scientifico (IRCCS) Fondazione Stella Maris, Pisa, Italy

^6^Department of Translational Research and of New Surgical and Medical Technologies, University of Pisa, Pisa, Italy

^7^Neonatal Intensive Care Unit, Children's Hospital A. Meyer, Florence, Italy.
